# The Right Not to Know: some Steps towards a Compromise

**DOI:** 10.1007/s10677-020-10133-9

**Published:** 2020-10-29

**Authors:** Ben Davies, Julian Savulescu

**Affiliations:** 1 Uehiro Centre for Practical Ethics, University of Oxford, Oxford OX1 1PT, UK

**Keywords:** Autonomy, Right not to know, Mill, Kant

## Abstract

There is an ongoing debate in medicine about whether patients have a ‘right not to know’ pertinent medical information, such as diagnoses of life-altering diseases. While this debate has employed various ethical concepts, probably the most widely-used by both defenders and detractors of the right is autonomy. Whereas defenders of the right not to know typically employ a ‘liberty’ conception of autonomy, according to which to be autonomous involves doing what one wants to do, opponents of the right not to know often employ a ‘duty’ understanding, viewing autonomy as involving an obligation to be self-governing. The central contribution of this paper is in showing that neither view of autonomy can reasonably be said to support the extreme stances on the right not to know that they are sometimes taken to. That is, neither can a liberty view properly defend a right not to know without limits, nor can a duty view form the basis of an absolute rejection of the right not to know. While there is still theoretical distance between these two approaches, we conclude that the views are considerably closer on this issue than they first appear, opening the way for a possible compromise.

## 1 Introduction

It is widely accepted that patients have a defeasible “right to know” information that is relevant to their own health (Council of Europe 1997: Article 10.2; Wilson 2005; Knoppers 2014). There is also institutional support for a “right *not* to know” (RNTK) including from international institutions (World Medical Association 1981, reaffirmed 2015; Council of Europe 1997, Article 10.2; UNESCO Office in Brazil 2000, Article 5c), i.e. a right held against medical professionals that they not disclose medical information to a patient in certain circumstances. While disagreement about a right to know concerns its boundaries, the RNTK is subject to a much deeper disagreement over whether it exists at all.

The RNTK is most commonly discussed in relation to genetics. Andorno (2004: 435) imagines Peter, a study participant who rejects the opportunity to be informed whether he has mutations that may cause Alzheimer’s. While some might insist that those running the study have a duty to inform Peter if he is at risk of ill health, proponents of the RNTK insist that Peter must be able to refuse such information, no matter how well-intentioned its provision. The RNTK has been raised in other areas too. Ndinya-Achola et al. (1995) argue for a RNTK HIV status, observing from a study of women who suffered serious negative effects after being discovered to be HIV-positive, such as being assaulted or even driven to suicide.

While one might understand the RNTK to apply even in the absence of relevant patient preferences - perhaps, for instance, vulnerable patients have a RNTK potentially very distressing information, which does not require consent - our focus in this paper is on a RNTK mediated by consent. On this understanding, while you have a right to receive medical information if you want to, you have a corresponding right not to receive it if that is your preference. This raises the question of what to do when patients express no preference, which we discuss in Section 3.

Despite some authors conflating them (Harris and Keywood 2001), a RNTK is not equivalent to a ‘right to ignorance’. A right to ignorance might imply that *anyone* who gives you unwanted knowledge violates your rights. The RNTK is typically held by patients against a much smaller group of individuals, such as those involved professionally with their care, or in public health research in which they are subjects (Nijsingh 2016; Morrisey and Walker 2018: 27). In other words, a RNTK is a right that *specific individuals* do not tell you things that you wish not to know, held *in a specific institutional context*. It is undoubtedly true that many who would exercise a putative RNTK would do so out of a desire to remain ignorant. However, this is not the only possible motivation for such a right: one might prefer to hear certain information from family rather than a medical professional, or to control the time at which one receives unwelcome news. Nonetheless, we focus on cases where the primary motivation is to remain ignorant.

With this narrower scope, it would not violate Jane’s RNTK if her father, John, were to inform her of his own recent diagnosis of a genetic disease such as Huntington Disease, giving Jane information about her own risks. This is because Jane’s RNTK is held only against specific individuals such as her doctor, not against the world in general. As one of us has argued Davies (2020), a RNTK that is understood in this way - i.e. as held in an institutional context only against medical professionals - is consistent with the claim that patients have an obligation not to remain ignorant about their health status. Patients may do something immoral by remaining ignorant; but this does not undermine their right to control the information given to them by medical professionals.

The RNTK has been defended and criticised on the basis of several different values, including privacy (Laurie 2014) and the patient welfare (Ndinya-Achola et al. 1995; McGleenan 1997: 44; Hallowell 1999; Bortolotti and Widdows 2011; Bullock 2016). Perhaps the primary point of contention in this debate, however, is around the justificatory role of autonomy. Appeals to autonomy have been used by both defenders and critics of a RNTK, suggesting a deeper divide over the value and role of autonomy in our lives. This paper begins in Section 2 by outlining a central argument against the RNTK that is based in autonomy, and then considers the response from defenders of that right.

The central contribution of the paper is in engaging with two distinct positions on autonomy which have been employed to defend strong stances respectively for and against a RNTK. These are the ‘liberty’ approach, based on the work of John Stuart Mill, which sees autonomy as a matter of doing what one prefers; and a ‘duty’ approach, thought by some proponents to derive from Immanuel Kant’s work, which frames autonomy as a duty of self-government. We show that neither view of autonomy can reasonably be said to support the extreme stances on the RNTK that they are sometimes taken to.

Some have used a strong liberty view of autonomy to defend the idea that individual choice is fundamental in all cases to whether a person acts autonomously. As we discuss in Section 2, this is often taken to provide considerable support to the idea that a RNTK is essential to the defence of autonomy. We note in Section 3, however, that even such an undemanding notion of autonomy requires that individuals understand the choices they are making to some extent.

Section 4 then turns to the duty view of autonomy, which some authors have used to support an equally strong view of the opposite kind, namely that we always have a rational obligation to acquire knowledge that is relevant to our life choices, and thus no RNTK. Against this view, we show that it relies on either an implausibly binary understanding of autonomy as something one either has or lacks entirely, or on the implausible view that we must always maximise our autonomy.

While real differences remain between the duty and liberty approaches, our conclusion is that on the issue of a RNTK, there is less distance between plausible versions of the views than has previously been thought. Thus, there may be greater scope for a compromise on this issue.

## 2 Autonomy and the RNTK

One central objection to a RNTK appeals to the value of autonomy. According to this objection, deliberately avoiding information that is medically relevant amounts to deliberately restricting the basis on which one makes choices that are important to how your life goes. Crucially, ignoring such information undermines the very possibility of acting autonomously. Such an argument has formed a major part of several influential papers that reject the RNTK.

For instance, as Robertson and Savulescu (2001: 42) put it:

Autonomy is self-government or self-determination. Being autonomous involves freely and actively making one’s own evaluative choices about how one’s life should go. Evaluative choice requires holding true beliefs.

Similarly, Rhodes (1998: 18) argues that:

From my point of view as an individual autonomous agent … when I choose to remain ignorant of relevant information, I am choosing to leave whatever happens to chance. I am following a path without autonomy. Now, if autonomy is the ground for my right to determine my own course, it cannot also be the ground for not determining my own course.

And Harris and Keywood (2001: 421) suggest that:

Ignorance of crucial information is inimical to autonomy in a way that other autonomylimiting choices are not. For where the individual is ignorant of information that bears upon rational life choices she is not in a position to be self-governing.

According to such arguments, the appeal to autonomy to ground a RNTK is incoherent, because the possession of relevant information is required for autonomous living. As we have suggested above, while we should not interpret the RNTK *as* a right to ignorance, we focus on cases where this is the primary motivation.

Such an argument could take a weaker or a stronger form. In its weaker form, this argument merely amounts to a refutation of *defences* of the RNTK that are themselves grounded in autonomy. Consider Rhodes’ claim, above, that “if autonomy is the ground for my right to determine my own course, it cannot also be the ground for not determining my own course”. This version of the argument is consistent with alternative justifications for the RNTK (e.g. by appeal to privacy) being successful. In that case, we might say that although autonomy is undermined by the RNTK, that right is all things considered justified because there are more important things than autonomy. For instance, one might think that people have a right to forgo autonomy, or that considerations of a patient’s well-being may trump the value of their autonomy in some cases.

A stronger version of the argument rejects this claim, aiming not only to defeat a particular justification for the RNTK, but to directly argue against it through the appeal to autonomy. This view requires both that autonomy is undermined by insisting on not knowing medical information, *and* that no other considerations typically outweigh this. This might be because autonomy is a ‘master-value’, primary over other possible considerations; or simply because in most central instances, the weight of other values involved does not tip the balance.

Supporters of the RNTK respond that one can make an autonomous choice to reject information. One way of interpreting this response is that while one’s ignorance might undermine or weaken the autonomy of a particular decision (i.e. one’s decision about one’s treatment), the separate decision *to remain ignorant* can nonetheless be autonomous (Andorno 2004; Herring and Foster 2012). Where knowledge might bring a person harm as well as good, or where the benefits are uncertain, autonomy might seem to demand that it is *up to them* to judge whether the value of choosing autonomously about their health outweighs the risks (Takala 2019). Such a view relies on a distinction between respecting and promoting autonomy. To respect a person’s autonomy requires respecting individual decisions, whereas to promote a person’s autonomy involves acting so as to ensure (at least) that she can act autonomously in the future.

Alternatively, one might understand the response as rejecting any inconsistency between a RNTK and autonomy at all. Häyry and Takala (2001) consider two views of autonomy. One of these views is derived from the views of John Stuart Mill, according to which we have the right to decide as we please - including irrationally - on issues that do not harm others. The second is (more loosely) inspired by a consideration of the philosophy of Immanuel Kant (see, particularly Rhodes’ (1998: 16-19) explicit appeals to Kant in justifying her stance), and posits a notion of autonomy as a duty to make informed decisions, based on a more fundamental duty of self-respect.

There are clear complexities in basing contemporary views on the work of historical philosophers. As an anonymous reviewer points out, there is some considerable distance between the *Kant-inspired* view outlined above and Kant’s actual work on autonomy. Thus, naming these views ‘Kantian’ and ‘Millian’ is apt for confusion. Rather, we call the Kant-inspired view, with its focus on autonomy as an obligation, the ‘duty’ view, and the Millian approach (which adheres more closely to its namesake’s actual views), the ‘liberty’ view. We refer to their respective proponents as ‘duty’ and ‘liberty’ theorists.

Assuming that proponents and opponents of a RNTK are indeed adopting significantly different models of autonomy, what does this tell us about the debate? We suggest that neither model straightforwardly supports its associated position on the RNTK.

## 3 The Liberty View

We begin with the liberty model broadly assumed by defenders of the RNTK, beginning with an observation made by Ost (1984: 305), that the central example employed by Mill himself (Mill 1859/2006: 165) to exemplify his position offers a more complex injunction than merely to leave people alone. Mill’s example involves a traveller who is about to cross a bridge that you know to be unsafe. Mill’s view is that while you may restrain the traveller to some extent in order to make sure that he is aware of the danger, once you know that he *is* aware, you may not impede him any further, even if you think his behaviour is foolish. Ost draws from this the conclusion that what we have called the ‘liberty view’ of autonomy cannot ground a RNTK, at least if such a view is to be directly derived from Mill. For even if a liberty– in contrast with a duty - approach gives us leeway to make foolish decisions, it apparently cannot give us the right to make *uninformed* decisions. Indeed, this gains support from Mill’s own explanation of the case; as he puts it, “liberty consists in doing what one desires, and [the traveller] does not desire to fall into the river”. In other words, the traveller does not *really* want to cross the bridge, because he does not desire the necessary consequence of doing so. Similarly, we might think, ignorance of illness might lead you to make choices whose consequences go against your desires.

Put this way, two of the three arguments canvassed at the beginning of this paper might be classified as broadly liberty-based. While Harris and Keywood do suggest that there may be a duty to know in some cases (e.g. where one’s ignorance might lead to harming others), their basic argument against a RNTK is conceived in terms of effective action: there are some pieces of information where ignorance of them blocks effective pursuit of one’s desires. Similarly, Robertson and Savulescu (2001) suggest that knowledge is required in order to make evaluative choices. The logic is similar to Mill’s. A patient who expresses a desire not to know some piece of medical information clearly expresses a desire. But, goes this line of argument, we may also assume that she has other desires, whose pursuit will be made more difficult if she lacks key pieces of knowledge.

However, things are more complex than this. First, we need to distinguish between two separate types of decision that patients might make. There are decisions that *use* medical information, e.g. decisions that are either directly about one’s health, or about one’s broader life in the context of health-related information.^1^ Call these ‘practical’ decisions. Clearly, a patient who refuses information about her health cannot make fully informed decisions of this type.

However, there are also decisions *about* information, e.g. whether to receive some piece of information. Call these ‘informational’ decisions. When we consider this distinction, it becomes clear that it is possible for a patient to make an informed decision to remain uninformed. If you know that your family has a genetic history of Huntington’s Disease, you can become very well informed about what Huntington’s would involve, and what risks you would take in refusing a personal diagnosis. While an autonomous decision about whether to find out whether you have Huntington’s requires *some* information, it clearly does not require information about whether you have the disease. However, it may also be true that your decision not to find out whether you have Huntington’s will reduce your autonomy with respect to certain practical decisions, such as long-term future plans. It seems possible to make a fully autonomous *informational* decision that will predictably reduce your autonomy in making certain future practical decisions. Simply noting that refusing the information will reduce your future autonomy, then, does not capture the full picture with respect to autonomy.

There are various reasons that one might choose to remain somewhat uninformed. For instance, one may need to know the risks and benefits of marriage before making an autonomous decision about marrying a particular person. But one does not need to know precisely how happy one will be with that person, even if this additional information might make your decision about whether to marry them more autonomous. This is partly because it is reasonable to want surprises, and partly because this happiness is in the making.

Matters are, of course, very different with respect to a disease like Huntington’s; the fact that one actually has the disease would clearly be an unwelcome surprise, whereas at least some surprises in a marriage will hopefully be welcome. Nonetheless, some potential sufferers may prefer to remain less than fully informed, because this frees them to live their life in hope, without the prospect of their disease hanging over them. For such patients, it may be enough to know that they are at 50% risk of Huntington Disease (since this allows for some preparation), without precisely knowing whether they will get it or not. Two options may be equally worthy of choosing even if one is informationally less rich; when faced with lives of comparable desirability, but which are mutually exclusive, we may best respect a person’s autonomy by allowing them control over how much information to receive. If they choose the less informationally-rich path, some subsequent decisions might be less autonomous than they could have been; and yet the original decision to remain in partial ignorance might nonetheless have been autonomous, and worthy of respect.

Second, while Mill’s example does seem to support the idea that autonomy rights do not stretch to the refusal of certain kinds of information, this does not necessarily speak against a RNTK. Assuming that we find Mill’s take on his own example plausible - i.e., we agree that we ought to interfere with the traveller in order to tell him that the bridge is unsafe, but no further if he acknowledges the danger and wants to cross anyway - we would then need to consider what it is about this information that makes it so important to divulge. One interpretation of Mill’s specific explanation of his case is that it is safe to assume that the traveller does not want to fall in the river; but more than this, it must be that we are permitted to assume that his wish not to fall in the river is stronger than his desire not to be “seized”. In other words, our interpretation of this case is that Mill appeals here to something like hypothetical consent.

On this interpretation, it is not the fact that the traveller’s decision to cross is uninformed which creates a permission to do something to him that is against his immediate desires (i.e. the desire not to be seized); rather, it is that we can safely assume that he has *dispositional* desires which he holds more strongly (i.e. not to fall in the river). It is rather less clear that this assumption holds in all cases of medical knowledge in a way that uniformly opposes the RNTK. To be analogous with Mill’s case, we would have to be confident that a patient (i) did not desire the various consequences that would occur if she continued without knowledge of her condition, but which could be avoided if she were to know, and (ii) that this desire was *stronger* than her desire not to know about the condition.

Neither of these is inevitable. A patient with a serious genetic condition might feel unable to have children upon diagnosis. She might also know this fact about her own character and, thus, prefer to remain in ignorance given her strong desire to have children. A further possible disanalogy with Mill’s case is that, since the traveller is about the embark on the crossing, our telling him is a *now or never* issue. In contrast, it is rarely, if ever, the case that someone must be given some medical information ‘now or never’.

However, the scope of this response should not be overstated. Imagine that, contrary to how most people would behave, you decide to ask Mill’s traveller whether he wants to know about the safety of the bridge. In asking the question, conversational context unavoidably offers some information; because while it is strange to ask someone if they want to know if the bridge they are about to cross is safe, it is stranger still to ask it when you know that the bridge *is* safe. As such, the asking does the telling. To some extent, the same is true in medicine. Supporters of a RNTK might insist that patients have a right to be asked whether they want to know a piece of medical information. But asking the question will often give some information; a doctor cannot ask about every possible medical risk, and so in asking whether a patient wants to know about *this* risk, she gives him information that a particular medical issue is on her radar.

We can link this point with an earlier observation about the distinction between practical and informational decisions. Our example involved an individual who already had considerable information (i.e. they knew their family history, and in that particular case their rough odds of having the condition). They were therefore capable of taking an informed informational decision about whether to receive information relevant to further practical decisions. But proponents of a RNTK might want to make a stronger claim, that autonomy entitles us to refuse information even when that informational decision is *itself* uninformed - or at least, far less well informed that our Huntington’s example. For instance, we can imagine a patient who is informed by her doctor that ‘something has come up in your test results’ that the doctor would like to investigate further. Of course, the patient could theoretically imagine each possible outcome, consider the potential consequences, and make a somewhat informed decision not to receive further information. But this is practically beyond even most medical professionals, let alone those who lack relevant knowledge and expertise. A patient who refused information at this stage could not, in our view, be said to be making even a minimally informed decision.

However, this does not mean that the standard of information is quite as high as that outlined in our initial case of the patient who may have Huntington’s. Huntington’s is unusual in that knowledge of a parents’ diagnosis gives a strong indication of one’s own risk. Since Huntington’s is a single-gene disorder, a parental diagnosis gives a secure probability of 50% that the patient will also develop the condition. But it would be a mistake to draw from this the conclusion that patients can only choose autonomously if they have *very precise* knowledge of the grounds of their risk, a *reasonably clear* probability indicator or risk, or indeed a *fairly high* probability of developing a condition. In many cases, patients can consider risk in a useful way by being told of medical concerns at a more general level. Here we offer two examples.

First, a doctor might suggest a number of possible explanations for a patient’s symptoms and include details of potential treatments (if any) and possible effects on quality or length of life. The patient can then decide if there are any conditions that she would not want to be informed of out of the list of candidates. Of course, some ways of doing this will not be successful strategies for remaining ignorant. If a patient says that she is happy to receive a diagnosis in all cases but one, then failing to receive a diagnosis will provide her with unwelcome information.

Second, doctors can often provide a generic list of possible conditions for which patients in a particular situation might want to be tested. During pregnancy, parents are offered the option of several standard screenings, including for Down’s syndrome, which they can refuse. Since screening is offered in all cases of pregnancy, the offer provides no unwanted information.

Of course, such idealised circumstances will not be available in all cases. Sometimes only one condition will present itself as an explanation of symptoms and offering a patient the opportunity to test for this condition will give them risk-related information.

We grant that there is potential value to having the right to control one’s life even in the absence of much information. For instance, such a right may be usefully held against overreach by governments. In a medical context, granting patients an enforceable authority to control receipt of information in all cases might be justified as protection against paternalism by medical professionals. Such an argument might go as follows: where there are limits on such rights of informational control, medical professionals may misjudge when it is appropriate to share or withhold information; may fail to elicit patients’ preferences on the receipt of information; or may ignore such preferences. Giving patients an absolute right of control over their data (that is, both a right to demand and a right to refuse relevant information) that is enforceable against medical professionals in an institutional context means that all of these are far less likely, since medical professionals would have an institutional obligation to listen to patients. Even if this means that patients sometimes make wholly uninformed – and, hence, non-autonomous – decisions, this might be a price worth paying to avoid the potential abuse and misuse of informational authority by medical professionals.

The immediate justification for such a right would not be autonomy, but a risk-averse strategy against what is often called ‘domination’ (Pettit 1997; Skinner 2008; Laborde 2010). In the language of civic republicanism, domination involves being subject to the arbitrary power of another, even if this power is never actually used. A policy where patients have an absolute veto on receiving information - even if that decision is uninformed, and even if it means that important future decisions are uninformed - might be justified as a robust defence against patients being dominated by health care professionals, on the grounds that only such an absolute veto could adequately protect against the possibility of, say, coercion by those with greater social power.

Still, such an argument might provide an autonomy-based ground for the liberty-theorist to insist on an absolute RNTK, if interpreted in a particular way. Non-domination is a view of what liberty requires; since liberty theorists equate autonomy with liberty (doing what one prefers), they might claim that we should understand this in civic republican terms, as the absence of domination. Thus, they might say, respect for autonomy implies absolute protections against domination.

In our view, however, such an argument is excessively cautious, and would hamper patients’ abilities to plan their own lives to an extent which would outweigh any benefits in terms of domination. While the decision that such a right makes possible - to refuse information even without knowing anything about what that information might concern - could in principle be autonomous, it would have a debilitating effect on any future attempts to make autonomous decisions.

An extreme liberty theorist may insist that if this is what the patient desires, then this is what respect for autonomy requires. But this ignores the potential impact on patients who do not have such extreme preferences. As we discussed above, certain questions have inherent informational potential. Thus, such an absolute right would potentially constrain doctors from performing even quite basic institutional functions, with a negative impact on many patients’ autonomy. While we do not claim that the argument we have just considered can be automatically dismissed, we do not think there is a good case for enshrining it at an institutional level.

On balance, then, liberty-theorists should accept that there are *some* requirements before a patient can be said to autonomously reject access to information that it is her right to have. As Mill’s bridge case demonstrates, the patient must *at least* be aware that she is making a choice of a certain kind; without intervention, the traveller fundamentally misconceives the choice she is faced with when deciding whether to cross the bridge. A patient who rejects information about whether she has Huntington’s may make autonomous decisions because the realm of possibilities is suitably constrained by the knowledge she does have (i.e., her uncertainty is *with respect to Huntington’s,* not more general). But a patient who does not know, even in a minimal sense, what she is rejecting cannot make that choice autonomously. Liberty theorists should thus support limits on a RNTK in the form of requirements that patients at least know what they are refusing information about.

We have argued that it can be consistent with autonomy to reject some kinds of relevant information. There is one powerful objection to this claim. Imagine Mill’s traveller once again. This time, he knows that the bridge he is about to cross is unsafe – it is rickety and missing many boards. What he doesn’t know, however, is whether it will definitely hold his weight. But you do: you are an engineer and have just stress tested all the parts. You say to him,

“You know that bridge is unsafe. But I know whether or not it will carry your weight. Do you want to know?”

It seems bizarre to refuse this further information. Why might he refuse?

He might refuse because he is an extreme thrill seeker, like the freeclimber who summited El Capitan, Alex Honnold. He just values risky activities. But the traveller’s case is different - he either will fall or not. There is actually no probability, no chance. As Honnold makes clear in the film ‘Free Solo’ (Free Solo 2018), he does not attempt a free climb without much preparation and training, and always believes he can do it. While what Honnold does is inherently risky, the traveller takes risks that are not essential to the activity he is undertaking.

Alternatively, there might be some life-defining goal on the other side. Maybe it is his soulmate, without whom he does not value continued life. Perhaps the meaning of his life lies in crossing that bridge. The additional information doesn’t matter in the context of that life and its values.

Finally, it may be that what is on *this* side of the bridge is too appalling for him to bear. As in the previous case, he will be indifferent to the information about whether the bridge will support his weight, because he must attempt to cross either way.

This example, and the reasons our traveller might have to refuse certain information, will not apply in all medical cases involving a RNTK. Much medical information is itself probabilistic, so that patients are faced not with a choice between certainty and probability, but between more and less precise probabilities. On the other hand, there is not always a highly important goal obtained, or significant cost avoided, by resting with probabilities rather than certainties. When the RNTK is motivated by fear, that fear should be addressed if it can be. But if there is some genuinely good reason to remain with probabilities, rather than certainties, it may be autonomous to reject relevant information.

## 4 The Duty View

Let us begin by recapping the duty approach to the RNTK. Duty theorists do not think that autonomy requires that one is able to do whatever one wants. Rather, it requires that one acts *rationally*. Our duty to acquire information is grounded in the fact that, as individuals who are capable of exercising sovereignty over our own actions, the choice between knowledge and ignorance is a choice between exercising this sovereignty or not. From a duty perspective, autonomy is not simply a right, but a duty, because it is the core of our ethical nature. Refusing medically relevant information is wrong, because it leaves one *unable* to act autonomously.

This view thus sets out a binary approach to autonomy: either you accept relevant information and are able to act autonomously; or you reject information and are unable to act autonomously, at least with respect to decisions to which that information is relevant. For instance, in the passage quoted earlier Harris and Keywood suggest that when someone is ignorant of relevant information, she is “not in a position to be self-governing” (and not the less binary claim that she is able to self-govern *less effectively*). Rhodes similarly argues that in choosing ignorance I choose “to leave whatever happens to chance” (and not, e.g., that I allow chance to play a greater role).

We reject this binary view of autonomy. It is far more plausible to think that autonomy comes in degrees, that one person can be less autonomous than another without lacking autonomy altogether. We suggest below, however, that such an approach weakens the duty theorist’s argument. First, however, we will briefly outline why the binary approach to autonomy and knowledge – quite apart from its independent implausibility – creates a dilemma.

A broad challenge for a binary view of the relationship between autonomy and knowledge – you either have sufficient knowledge to be autonomous, or you don’t – is the issue of how much information you require to act autonomously. We can begin by ruling out as implausible an extreme view: that autonomy requires *complete* information. Complete information is impossible for any human being. Even if we were to somehow limit our claim to the need for complete ‘medical’ information, it is unlikely that even a medical professional could be said to have this. Information may be unobtainable because we do not yet have relevant evidence; because the patient does not have the relevant training to understand; or because the sheer quantity of relevant information is such that *some* facts must go unheard, unremembered or otherwise unknown.

Duty theorists must therefore offer a view of the type of information a patient needs in order to decide autonomously. The obvious choice seems contained in the above quote from Harris and Keywood, i.e. that the relevant kind of information is that which “bears upon” rational life choices, or which is “crucial”. Similarly, Rhodes (op. cit.) suggests we are obligated to learn information that is “likely to make a significant difference in my decisions”.

These caveats certainly serve to make more reasonable the claim that autonomy requires knowledge. But they still face a fundamental problem. Consider some piece of information that a duty theorist would plausibly claim is essential for genuinely autonomous choice, such as the information that one has a 50% chance of having the gene that gives you Huntington’s Disease.

Duty theorists face a dilemma about such information. Consider Elizabeth, who lived in the late nineteenth century, long before the possibility of discovering whether one had the genetic basis for Huntington’s. Elizabeth lacks the very same information as Joe, who lives today and refuses a Huntington’s-related diagnosis. If duty theorists insist that Joe lacks autonomy (in, recall, a *binary* sense) because he lacks this information, they must also decide whether Elizabeth lacks autonomy.

One position is to say that if a piece of knowledge is crucial to Joe’s autonomy, it is also crucial to Elizabeth’s. Since duty theorists insist that Joe is not autonomous, neither is Elizabeth. This implies that there was a wide variety of decisions that Elizabeth could not make autonomously, because she did not know whether she had the relevant genetic risk. Indeed, it implies that *nobody living prior to genetic knowledge about Huntington’s* was autonomous. This view coheres with the intuitive thought that advances in scientific knowledge have at least the potential to increase our control over our lives, and hence to increase the number of people who possess autonomy. But its implication that almost all people throughout history have lacked autonomy is hard to accept.

Additionally, it is not clear why the binary possession of autonomy should be indexed to information that *current* residents of *wealthy* countries can obtain. There are many pieces of medical information that are not currently obtainable by us, but which are relevant to our life choices. If we deny that Elizabeth could choose autonomously because of her lack of genetic information about Huntington’s, it is hard to see why it must not also be true that Joe is unable to act autonomously, given various facts about ourselves that current medicine cannot deliver.

A second option, then, is to note that Rhodes provides a caveat to her claim that autonomy requires information that will likely make a significant difference to decisions, accepting that this is only true if such knowledge is “obtainable with reasonable effort” (op. cit.). Precisely what makes knowledge reasonably obtainable is open for debate. It is clear that genetic knowledge is not obtainable for Elizabeth. Is it obtainable by someone living in a contemporary society without realistic access to the relevant technologies? Is it obtainable by someone whose scientific knowledge is such that they would not really *understand* genetic information that was given to them? We will not aim to settle such questions here, but only mark them as questions that a fully developed account of this kind must answer.

However we set the boundaries of knowledge’s effect on autonomy, though, this proposal also has an odd implication. If we compare Joe and Elizabeth, a view that indexes autonomy (in a binary manner) to the possession of ‘obtainable’ relevant information turns out to make it more difficult for Joe, the modern patient, to achieve autonomy than for Elizabeth, who lived in the nineteenth century. As a resident of the UK in the twenty-first century, Joe has potential access to a far greater wealth of knowledge than did Elizabeth. That means that Joe can know everything Elizabeth did, and more, and yet it be true that while Elizabeth was autonomous, Joe is not. On this view, then, advances in science will often reduce the number of people who are autonomous, because it will generate knowledge that is both relevant to and obtainable by them, but which they do not possess.

As we suggested above, the way forward is to note that autonomy is both context-specific and can come in degrees. We can agree that both the Joe and Elizabeth lack autonomy *to some degree* even if they avail themselves fully of all information that is accessible to them. However, Elizabeth likely lacks autonomy over a greater range of choices, and to a greater degree, than Joe does. Additionally, when Joe refuses a Huntington’s diagnosis, he makes himself less autonomous than he could have been.

Yet when we take this view of autonomy, the force of claims that refusing information means that we are not able to be self-governing, or that we are leaving things to chance, is significantly reduced. If autonomy comes in degrees, it is possible to accept a duty to exercise one’s capacity for autonomy to some extent without thereby thinking that this duty is a maximising one. A putative obligation to *maximise* autonomy would be extremely demanding, requiring that we dedicate ourselves to pursuing knowledge wherever possible, at the cost of other valuable aspects of life. Thus, one might choose to remain ignorant of some information, and yet still be *sufficiently* autonomous. A choice to remain ignorant of one’s Huntington status, for instance, might mean one is less autonomous, but sufficiently autonomous to govern one’s own life.

Assuming that duty theorists will not be content simply with concluding that some people are more autonomous than others, and will wish to still pursue the idea that there is an *obligation* relating to autonomy, our suggestion is that they should adopt a *satisficing* approach to the purported duty of autonomy: our obligation is to have sufficient control over our lives, or to acquire sufficient knowledge to be self-governing. Precisely where that line is to be drawn is a broader project than we can undertake here. Yet it will surely extend to a minimal level of information being provided to healthcare patients, including the fact that there is a choice to be made about receiving information about a particular condition. As readers will note, this is equivalent to our proposal in the previous section of what liberty theorists ought to accept.

Duty theorists may want to go further than this. However, if the duty to be autonomous is not a maximising one, this suggests that there must be at least a degree of patient discretion in deciding precisely which information to acquire. Precisely how large a degree that is depends on our understanding of the limits on the duty to be autonomous.

One possibility is to recognise that no decision can be perfectly autonomous, given inevitable limits on our knowledge, but to insist that every choice we make (or perhaps, every choice that will have a significant effect on our lives) must be sufficiently autonomous. This would mean that patients cannot reasonably reject information that will render future, practical decisions non-autonomous. However, it is not clear that the information that you *actually have* a particular condition, or even the relevant genes that place you at high risk of developing it, is always necessary to meet this criterion. Rather, it will often be enough that a doctor communicates their reasons for thinking that the patient may be at risk of a particular condition. Knowing that a disease is a relevant alternative will often be sufficient for patients to rationally incorporate that possibility into their life plans, particularly when there is some other important life goal or value at stake. If I know that I am at risk of a condition that will mean I never see old age, for instance, I then have the option of adjusting my spending habits to focus more on the present. However, if I am certain I will develop a serious disease in middle age, maybe I will not have the children I currently want very much.

Of course, my decisions might be even more rational if I knew more precisely the odds of my developing that condition. But it is not obviously true that my refusing information of the second kind (that I have a 70% chance of developing the disease), while possessing information of the first kind (that for various reasons my doctor regards my developing the disease as a realistic possibility) means that I am unable to make rational choices about my aspects of my future that would be affected by having this condition.

As we acknowledged in the previous section, it will not always be possible to distinguish these two types of information so easily. The information that my parent has developed Huntington’s disease tells me what my probability of developing the disease is, and thus it is hard to see how we could communicate to someone that Huntington’s is a ‘live option’ without also communicating their degree of risk. Nonetheless, there is then a similar relationship between knowledge of one’s degree of risk, and the potential knowledge that one actually has the Huntington’s gene. While testing for the gene would provide a greater degree of certainty, it is not obvious that knowledge of the 50% risk provides an insufficient basis to make autonomous choices with respect to the possibility of developing Huntington’s, particularly when certainty will close off important life options.

As such, we suggest that there is considerable space from within a duty view to allow for a RNTK, albeit one that has limits. Duty theorists who want to argue that there is *no* such right in a medical context must either insist on a very (and, in our view, implausibly) strong duty to maximise autonomy quite generally, or must explain why medical decisions are always such that we must maximise our autonomy in this field.

## 5 Conclusion

We have suggested that neither duty nor liberty views of autonomy justify the extreme stances on a putative RNTK that proponents sometimes take them to. While there are still clear differences between these views – most clearly in their understanding of autonomy’s nature and value, but also in their practical recommendations – our view is that both perspectives can accept a limited RNTK, when considered from the perspective of autonomy. A RNTK does not entail a right to be entirely ignorant, but neither does the value of autonomy imply a duty to maximise the control we exercise over our lives; hence, it is consistent with our rejecting some kinds of medical information in some contexts.

## Data Availability

Not applicable.

## Footnotes

1 Some proponents of a right not to know point to cases where a condition has no cure as examples where there is no obvious practical advantage to knowledge, e.g. Takala (2019: 227). For discussion see Hertwig and Engel (2016). But knowing that one has an incurable disease may still be useful; for instance, knowing that one has Huntington’s, and is thus very unlikely to survive to old age, makes a significant difference to one’s life plans. This is not to say that it is always best, all things considered, to have medical information; rather, benefits from knowledge are usually possible even in the absence of medical interventions.

## References

[R1] Andorno R (2004). The right not to know: an autonomy based approach. J Med Ethics.

[R2] Bortolotti L, Widdows H (2011). The right not to know: the case of psychiatric disorders. J M Ethics.

[R3] Bullock E (2016). Mandatory disclosure and medical paternalism. Ethical Theory Moral Pract.

[R4] Council of Europe (1997). Explanatory report to the convention on human rights and biomedicine.

[R5] Davies B (2020). The right not to know and the obligation to know. Journal of Medical Ethics.

[R6] Chin J, Vasarhelyi EC, Free Solo (2018). [Film].

[R7] Hallowell N (1999). Doing the right thing: genetic risk and responsibility. Sociol Health Illn.

[R8] Harris J, Keywood K (2001). Ignorance, information and autonomy. Theor Med.

[R9] Häyry M, Takala T (2001). Genetic information, rights, and autonomy. Theor Med.

[R10] Herring J, Foster C (2012). “Please don’t tell me”: the right not to know. Camb Q Healthc Ethics.

[R11] Hertwig R, Engel C (2016). Homo Ignorans: deliberately choosing not to know. Perspect Psychol Sci.

[R12] Knoppers B (2014). Introduction: from the right to know to the right not to know. J Law Med Ethics.

[R13] Laborde C (2010). Republicanism and global justice: a sketch. Eur J Political Theory.

[R14] Laurie G (2014). Recognizing the right not to know: conceptual, professional and legal implications. J Law Med Ethics.

[R15] McGleenan T, Chadwick R, Levitt M, Shickle D (1997). Rights to know and not to know: is there a need for a genetic privacy law?. The right to know and the right not to know.

[R16] Mill JS, Warnock M (1859/2006). On liberty. Utilitarianism and on liberty: including Mill’s ‘essay on Bentham’ and selections from the writings of Jeremy Bentham and John Austin.

[R17] Morrisey C, Walker RL (2018). The ethics of general population preventive genomic sequencing: rights and social justice. J Med Philos.

[R18] Ndinya-Achola J, Temmerman M, Ambani J, Piot P (1995). The right not to know HIV-test results. Lancet.

[R19] Nijsingh N (2016). Consent to epistemic interventions: a contribution to the debate on the right (not) to know. Med Health Care Philos.

[R20] Ost DE (1984). The ‘right’ not to know. J Med Philos.

[R21] Pettit P (1997). Republicanism: a theory of freedom and government.

[R22] Rhodes R (1998). Genetic links, family ties and social bonds: rights and responsibilities in the face of genetic knowledge. J Med Philos.

[R23] Robertson S, Savulescu J (2001). Is There a Case in Favour of Predictive Genetic Testing in Young Children?. Bioethics.

[R24] Skinner Q, Laborde C, Maynor J (2008). Freedom as the absence of arbitrary power. Republicanism and political theory.

[R25] Takala T (2019). Genetic moralism and health. Camb Q Healthc Ethics.

[R26] UNESCO Office in Brazil (2000). Universal declaration on the human genome and human rights: from theory to practice.

[R27] Wilson J (2005). To know or not to know? Genetic ignorance, autonomy and paternalism. Bioethics.

[R28] World Medical Association (1981). Declaration of Lisbon on the rights of the patient.

